# Endoscopic ultrasound-guided coiling and glue injection for bleeding from isolated rectal varices

**DOI:** 10.1055/a-2512-4927

**Published:** 2025-01-29

**Authors:** Yu Tang, Min Xie, Xiaoling Zhou, Junyi Zhuo, Xianfei Zhong

**Affiliations:** 1 Gastroenterology, The People’s Hospital of Leshan, Southwest Medical University, Leshan, China; 2 Health Care Center, The People’s Hospital of Leshan, Southwest Medical University, Leshan, China

A 73-year-old woman with hepatitis B-related cirrhosis was referred to our hospital with recurrent episodes of hematochezia. Computed tomography (CT) revealed the presence of the rectal–pelvic variceal plexus, as well as ascites ( Fig. 1 ). Colonoscopy identified isolated varices with stigmata of recent bleeding in the upper rectum ( Fig. 2 ). Endoscopic ultrasound (EUS) demonstrated that the rectal varices measured 20 mm in diameter and were connected to extraluminal varices via perforating veins ( Fig. 3 **a, b** ).

**Fig. 1:**
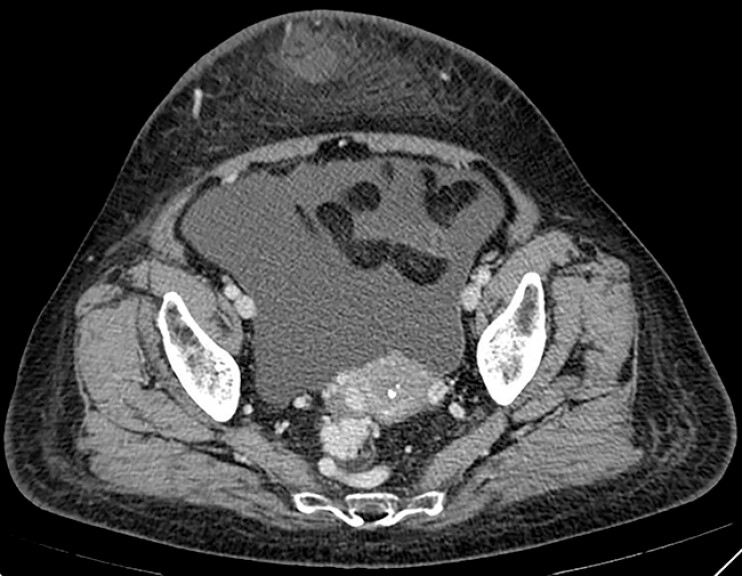
Contrast-enhanced computed tomography image showing the rectal–pelvic variceal plexus and ascites.

**Fig. 2:**
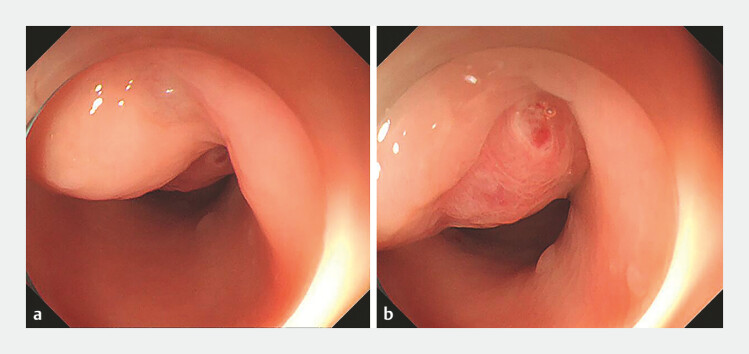
Colonoscopy images showing: **a** isolated varices; **b** stigmata of recent bleeding in upper rectum.

**Fig. 3:**
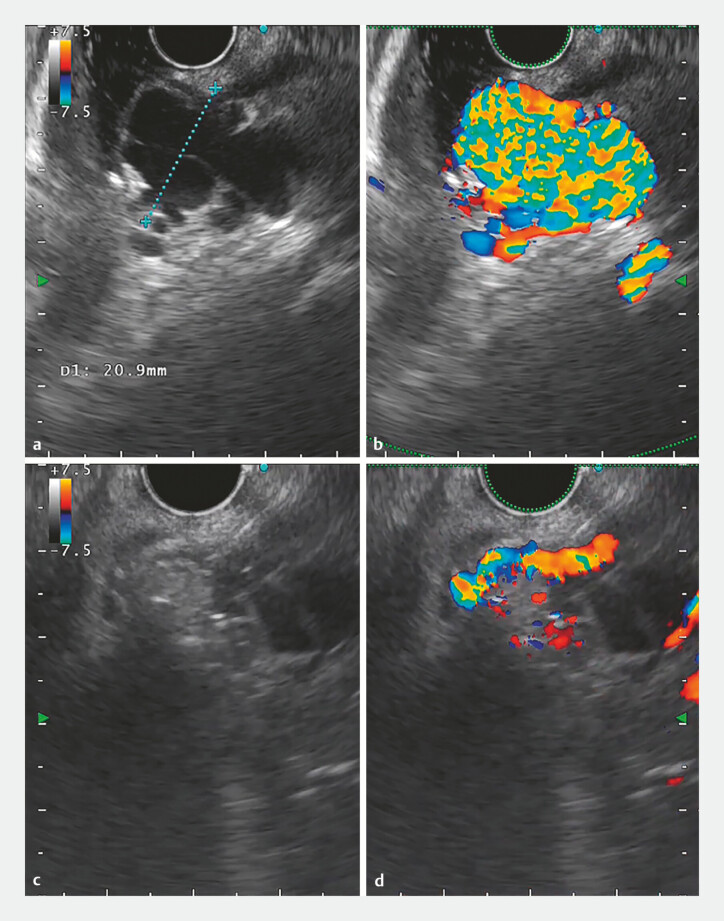
Endoscopic ultrasound (EUS) images showing the rectal varices: **a, b** prior to injection; **c, d** after coil deployment and subsequent glue injection, with a significant reduction in Doppler flow.

After the available therapeutic options had been discussed with the patient, she opted for EUS-guided coiling and glue embolization for initial hemostasis. Under EUS guidance, a 19-gauge needle preloaded with a 0.035-inch × 10-mm × 14-cm embolization coil (Nester, Cook Medical, Bloomington, Indiana, USA) was used to puncture the varices. After the coil had been deployed, 1 mL of cyanoacrylate glue and 1.5 ml of lauromacrogol were injected ( Video 1 ). A significant reduction in Doppler flow was observed, confirming the obliteration of the varices ( Fig. 3 **c, d** ). No adverse events were reported during or after the procedure. A post-procedural plain radiograph revealed the coil positioned in the pelvis ( Fig. 4 ). The patient did not experience any recurrence of hematochezia during a follow-up period of 6 months.

Successful treatment of bleeding from isolated rectal varices with endoscopic ultrasound-guided coiling and glue injection.Video 1

**Fig. 4:**
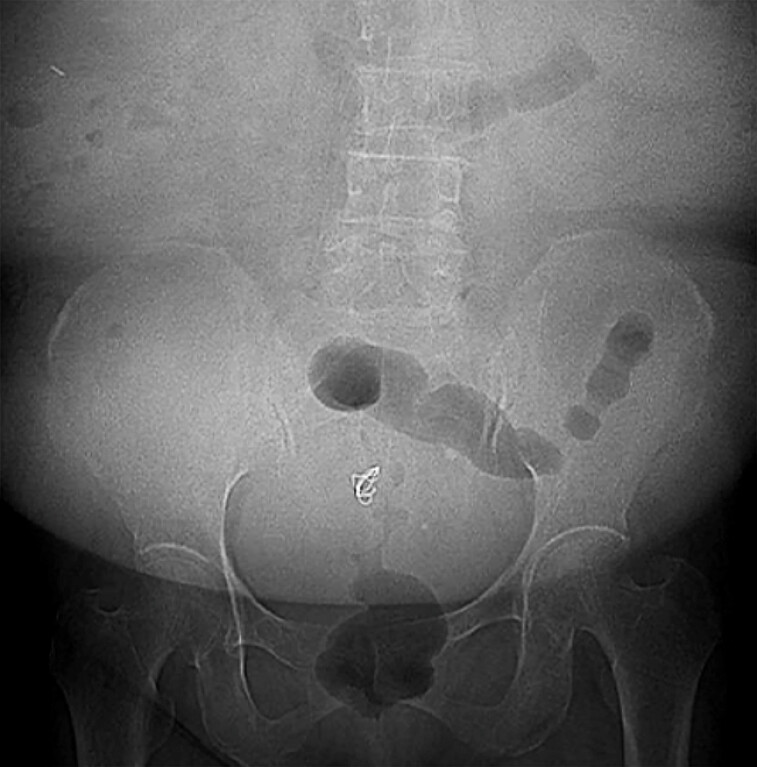
Post-procedural plain radiograph showing the coil in the pelvis.

There is currently no consensus regarding the optimal therapeutic strategy for managing hemorrhage from isolated rectal varices. Endoscopic glue injection alone guided by fluoroscopy has recently been recommended 1 . EUS not only facilitates precise intravariceal delivery of embolizing agents, but also allows for real-time monitoring of the therapeutic effect. The deployment of a coil serves as a scaffold for the glue, thereby reducing the volume of glue required and minimizing the risk of ectopic embolization 2 . Our findings demonstrate that EUS-guided coiling, in conjunction with glue injection, is a safe, effective, and straightforward approach to achieving initial hemostasis in cases of bleeding from isolated rectal varices.

Endoscopy_UCTN_Code_TTT_1AS_2AZ

## Notes

Endoscopy E-Videos https://eref.thieme.de/e-videos
*E-Videos* is an open access online section of the journal *Endoscopy* , reporting on interesting cases and new techniques in gastroenterological endoscopy. All papers include a high-quality video and are published with a Creative Commons CC-BY license. Endoscopy E-Videos qualify for HINARI discounts and waivers and eligibility is automatically checked during the submission process. We grant 100% waivers to articles whose corresponding authors are based in Group A countries and 50% waivers to those who are based in Group B countries as classified by Research4Life (see: https://www.research4life.org/access/eligibility/ ). This section has its own submission website at https://mc.manuscriptcentral.com/e-videos .
